# Supplementary material for: Euterpe oleracea Mart. Bioactive Molecules: Promising Agents to Modulate the NLRP3 Inflammasome
Source: Biology (Basel). 2024 Sep 17;13(9):729. doi: 10.3390/biology13090729 (PMC11428631; doi:10.3390/biology13090729)
Supplement: Supplementary file 1 [file biology-13-00729-s001.zip › biology-3172690-supplementary.pdf]

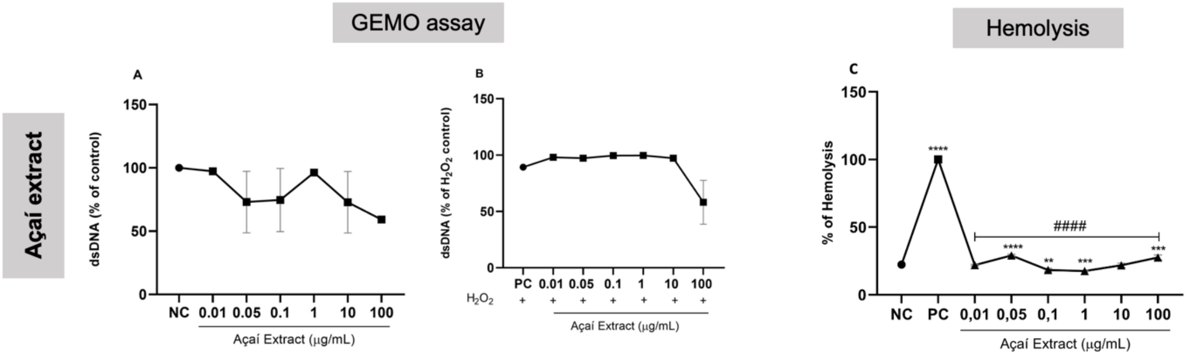

Figure S1. Açaí extract safety profile. **(a)** GEMO assay - Genotoxic effect. NC: dsDNA and 1X TE buffer. **(b)** GEMO assay - Genoprotective effect. PC: H_2O_2 3M. **(c)** Hemolysis. NC: red blood cells and 1X PBS buffer. PC: Distilled water. Statistical analysis was performed by One-way Anova followed by Tukey *post hoc*. Results with $p<0.05$ were considered significant. * Represents comparison to the negative control; # Represents comparison positive control. *$p<0.05$; **$p<0.01$; ***$p<0.001$; ****$p<0.0001$.

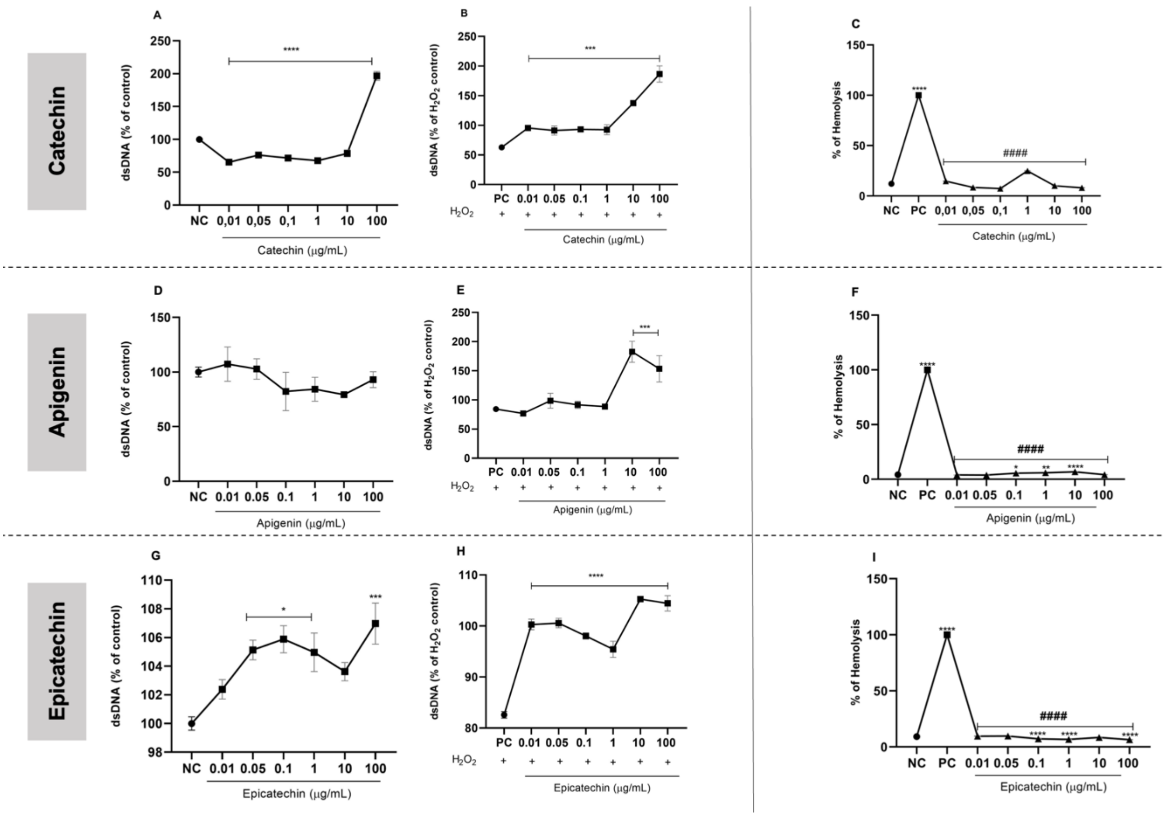

Figure S2. Bioactive molecules safety profile. **(a, d and g)** GEMO assay - Genotoxic effect. NC: dsDNA and 1X TE buffer. **(b, e and h)** GEMO assay - Genoprotective effect. PC: H_2O_2 3M. **(c, f and i)** Hemolysis. NC: red blood cells and 1X PBS buffer. PC: Distilled water. Statistical analysis was performed by One-way Anova followed by Tukey *post hoc*. Results with *p*<0.05 were considered significant. * Represents comparison to the negative control; # Represents comparison positive control. *$p<0.05$; **$p<0.01$; ***$p<0.001$; ****$p<0.0001$.

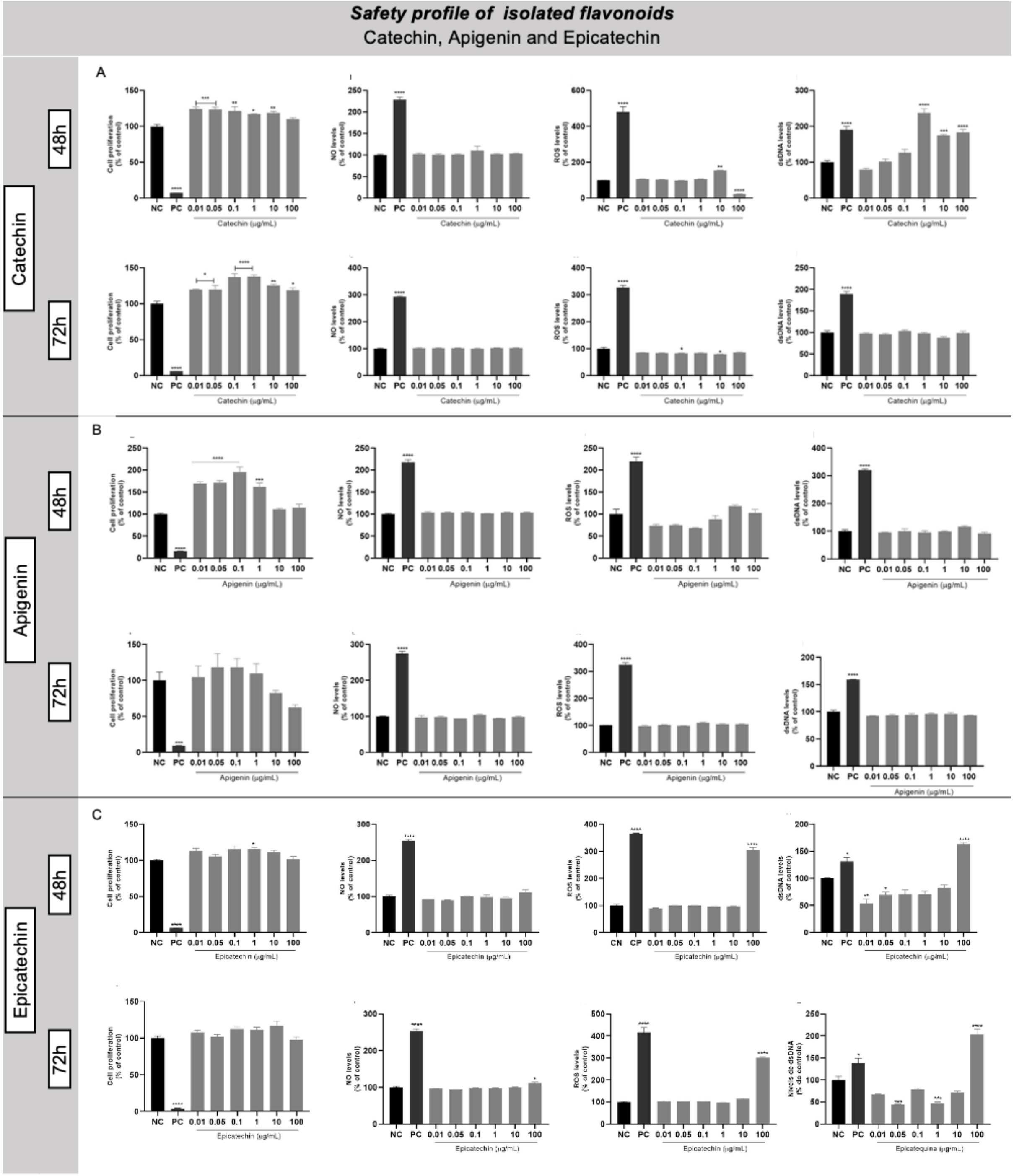

Figure S3. Bioactive molecules concentration-response curve - Safety profile evaluation. VERO cells were exposed to different concentrations of catechin, apigenin and epicatechin after 48 and 72h of incubation. Assessment of cell proliferation, levels of NO, ROS and extracellular dsDNA. NC: negative control (cells under conventional cell culture condition); PC: cells exposed to 200 µM of H_2O_2 for MTT, DCFH-DA and PicoGreen assays and 10 µM of sodium nitroprusside for NO determination assay; Statistical analysis was performed by One-way Anova followed by Tukey *post hoc*. Results with $p<0.05$ were considered significant. *$p<0.05$; **$p<0.01$; ***$p<0.001$; ****$p<0.0001$.
